# Super-compact universal quantum logic gates with inverse-designed elements

**DOI:** 10.1126/sciadv.adg6685

**Published:** 2023-05-26

**Authors:** Lu He, Dongning Liu, Jingxing Gao, Weixuan Zhang, Huizhen Zhang, Xue Feng, Yidong Huang, Kaiyu Cui, Fang Liu, Wei Zhang, Xiangdong Zhang

**Affiliations:** ^1^ Key Laboratory of advanced optoelectronic quantum architecture and measurements of Ministry of Education, Beijing Key Laboratory of Nanophotonics and Ultrafine Optoelectronic Systems, School of Physics, Beijing Institute of Technology, 100081 Beijing, China.; ^2^ Frontier Science Center for Quantum Information, Beijing National Research Center for Information Science and Technology (BNRist), Electronic Engineering Department, Tsinghua University, Beijing 100084, China.; ^3^ Beijing Academy of Quantum Information Sciences, 100193 Beijing, China.

## Abstract

Integrated quantum photonic circuit is a promising platform for the realization of quantum information processing in the future. To achieve the large-scale quantum photonic circuits, the applied quantum logic gates should be as small as possible for the high-density integration on chips. Here, we report the implementation of super-compact universal quantum logic gates on silicon chips by the method of inverse design. In particular, the fabricated controlled-NOT gate and Hadamard gate are both nearly a vacuum wavelength, being the smallest optical quantum gates reported up to now. We further design the quantum circuit by cascading these fundamental gates to perform arbitrary quantum processing, where the corresponding size is about several orders smaller than that of previous quantum photonic circuits. Our study paves the way for the realization of large-scale quantum photonic chips with integrated sources and can have important applications in the field of quantum information processes.

## Teaser

The smallest ever revolutionary optical quantum logic gates have been created on silicon chips using an inverse design method.

## INTRODUCTION

The universal quantum computer is a device capable of simulating any physical system and represents a major goal for the field of quantum information science, which can be obtained by networks of the quantum operators in universal gate sets, such as the controlled-NOT (CNOT) gate and single-qubit gates (*1*). Quantum photonic integrated circuits with CNOT gates and single-qubit gates are well recognized as attractive technology offering great promise for achieving large-scale quantum information processing (*2*–*9*). In recent years, many studies have been done to construct photonic quantum gates and integrated photonic circuits to perform quantum information processing (*4*–*7*, *10*–*24*). At present, the footprints of silicon photonic quantum circuits to implement arbitrary two-qubit processing constructed by multilayer Mach-Zehnder interferometers (MZIs) are on the scale of millimeters (*5*). It is still very difficult to construct a chip in this way to perform the complexity of quantum tasks because the number of quantum gates required increases exponentially with the increase of the quantum-state complexity to simulate an arbitrary *n* qubit quantum information process. This requires one to integrate many photonic components on an ultra-compact chip, and thus, it is extremely important to reduce the size of photonic components. Until now, the previous works report the realization of the plasmon-based (*19*) and symmetry-breaking waveguide–based (*20*) quantum CNOT gates, whose footprints are about ~200 and ~21 μm^2^. Thus, how to construct optical quantum logic devices with extremely smaller sizes on the chip becomes an open problem.

On the other hand, recent investigations have shown that some inverse-designed methods can display various advantages in the design of compact optoelectronic devices (*25*–*43*), and many basic elements have been designed, including wavelength demultiplexers (*27*), polarization beam splitters (*28*), and so on. These inverse-designed devices have better performances and more compact structures than those based on traditional design methods. For example, inverse-designed metastructures that solve equations have been demonstrated (*29*, *30*), and an on-chip integrated laser-driven particle accelerator has been realized by the inverse design (*31*). All these studies focus on the devices in the classical electromagnetic wave systems and bring the great achievement of device miniaturization and high performance. In addition, the single-photon source has been theoretically designed by the inverse design method (*32*). As for the photonic quantum logic devices, inverse design methods have not been used. It is meaningful to ask whether smaller footprints and fewer losses could appear when the inverse-designed method is applied to the design of quantum logic devices on the chip.

In this work, we design and fabricate super-compact universal quantum logic gates using the inverse-designed method on a silicon photonic chip with an integrated source. The sizes for the fabricated optical CNOT gate and single-qubit gates (Hadamard gate) on the chip are the smallest optical quantum gates ever verified in the world. Moreover, a further extension of the arbitrarily photonic quantum circuit is also provided by combining a number of CNOT and single-qubit gates.

## RESULTS

### The inverse-designed single-qubit gate

**Fig. 1.**
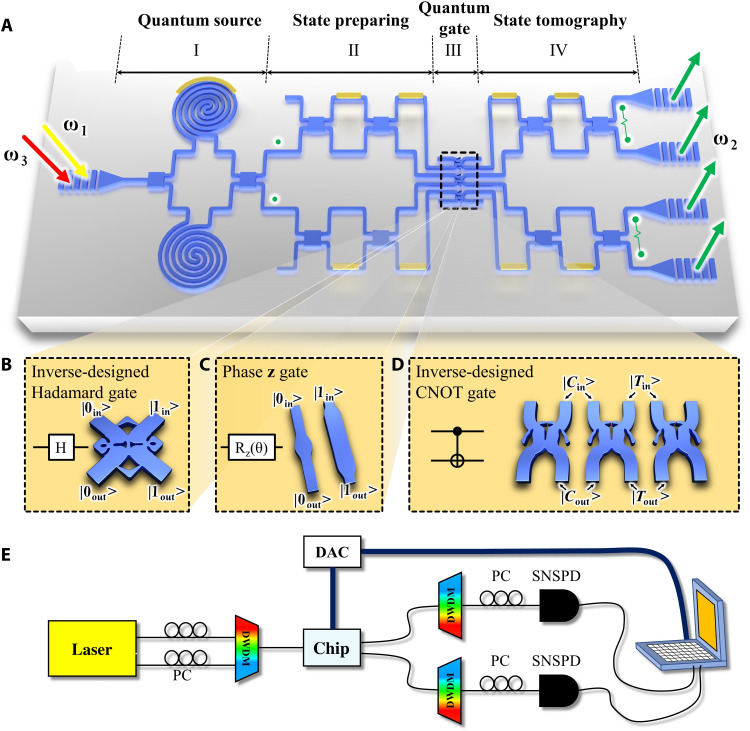
The inverse-designed super-compact quantum logic gates. (**A**) Schematic diagram of the photonic chip to measure the quantum gates. (**B**) Inverse-designed Hadamard gate. (**C**) Phase z gate. (**D**) Inverse-designed CNOT gate. (**E**) Schematic diagram of the experimental setup. PC, polarization controller; DAC, digital-to-analog converter; SNSPD, superconducting nanowire single-photon detector.

The quantum chip is designed on the silicon-on-insulator (SOI) platform with the 220-nm-thick Si layer. The schematic diagram is shown in Fig. 1A. There are four modules: (i) quantum source, (ii) state preparing, (iii) quantum gates, and (iv) state tomography, from the left to the right on the chip. In this work, we focus on the inverse-designed super-compact quantum gates, including the Hadamard gate, the phase z gate, and the CNOT gate, as shown in Fig. 1 (B to D, respectively). The size of each area in the quantum chip can be found in section S1.

Now, let us first introduce the corresponding experimental setup to test these gates, as indicated in Fig. 1E. The two continuous-wave pump lasers of 35 mW (the frequencies are ω_1_ = 194.9 THz and ω_3_ = 195.3 THz, and the corresponding wavelengths are 1538.19 and 1535.04 nm) are combined and injected into the experimental system. By the polarization controllers, the polarization of the pump lasers can be adjusted to be injected into the chip with the maximum power by the one-dimensional (1D) transverse electric grating coupler. As shown in the left of Fig. 1A, when the pump lasers (marked as the red and yellow arrows) enter module I, it is equally split into the upper and lower waveguides by the multimode interference (MMI) coupler. Here, the spontaneous four-wave mixing (SFWM) process is stimulated in two 6-mm-long silicon waveguides, and two photons are generated at the central frequency ω_2_ = 195.1 THz (2ω_2_ = ω_1_ + ω_3_), whose corresponding wavelength is 1536.61 nm. We use the thermally tuned phase shifters (the heater made by the titanium electrode) to adjust the phase difference between two waveguides of the photon source, as marked in yellow on the Si waveguide. Thus, the antibunch state (two photons at two different waveguides) can be obtained after the second MMI. In the second module, there are two arbitrary path-encoded quantum state generators made by two MZIs and four phase shifts (PSs). Thus, the two path-encoded arbitrary single qubits can be generated by controlling four heat electrodes and injected into the inverse-designed quantum gates (the third module). The output quantum qubits are projected and detected by the fourth module of the state tomography, which is the same as the second module and made by MZIs and PSs. After going through four modules of the quantum chip, the quantum states are coupled into the fibers by 1D gratings and detected by the fiber-coupled superconducting nanowire single-photon detectors (SNSPDs). In addition, two cascaded dense wavelength division multiplexers (cascaded DWDMs) are used to remove the residual pump photons. Nine thermal electrodes on the chip also require a computer-controlled digital-to-analog converter for voltage controlling. On the basis of such a setup, the functions of designed quantum gates can be tested by analyzing the measured two-photon coincidence counts (see Methods for more details).

Here, we primarily consider the design process of the quantum gates. The first one is to design the Hadamard gate, which is one of the fundamental single-qubit gates. Generally, the 50:50 beam splitter (BS) is needed to realize the Hadamard gate on a photonic quantum chip, where the input quantum state ∣ϕ〉 (coming from the input port *a*_in_) is taken as superposition state *c*_1_∣ϕ_1_〉 + *c*_2_∣ϕ_2_〉. Here, *c*_1_ (*c*_2_) represents the coefficient of the output state ∣ϕ_1_〉 (∣ϕ_2_〉) from the output port *a*_out_ (*b*_out_). To carry out the optimization process, we first define an associated objective function of the BS with the single-photon excitation, ${\text{Γ}}_{\lambda }=c_{1}^{2}+c_{2}^{2}$. During the design process, the objective function is maximized and the optimized structure appears. This process can be described by the following equation

$$\underset{\varepsilon_{{\mathrm{sio}}_{2}}\leq \varepsilon (r)\leq \varepsilon_{\mathrm{si}}}{\max}\text{Γ}=\sum_{\lambda }{\text{Γ}}_{\lambda }[\varepsilon (r)]$$ where ε(***r***) ∈ [ε_sio_2__, ε_si_] is the design field, which represents the material distribution of permittivities (between SiO_2_ and Si), and λ is the wavelength, which is summed in the objective function with three different values (λ = 1520, 1550, and 1580 nm) to extend the range of operation frequencies. Moreover, the additional condition, $\gamma_{1}\leq c_{1}^{2}/c_{2}^{2}\leq \gamma_{2}$ (γ_1_ and γ_2_ are optimization parameters), should be added to limit the squares of amplitudes of output quantum states from two ports becoming nearly identical, so that the splitting ratio of 50:50 can be realized (see section S2 for detailed optimization procedures). In addition, we also do a sensitivity analysis of our device, which ensures a good performance even if manufacturing deviations exist. We use the double filtering method (*44*, *45*) in the inverse-design process. In brief, the double filtering method consists of applying the filter and threshold procedure twice on the design field, where, in the second application, three different threshold values are applied to obtain three different realizations of the design fields corresponding to under-etching (over-etching). Thus, the over- and under-etching cases are simultaneously optimized. In such a way, we can get a robust device against the manufacturing defect of under-etching (over-etching), which is the most common defect in optical chip fabrication (see section S3 for details).

**Fig. 2.**
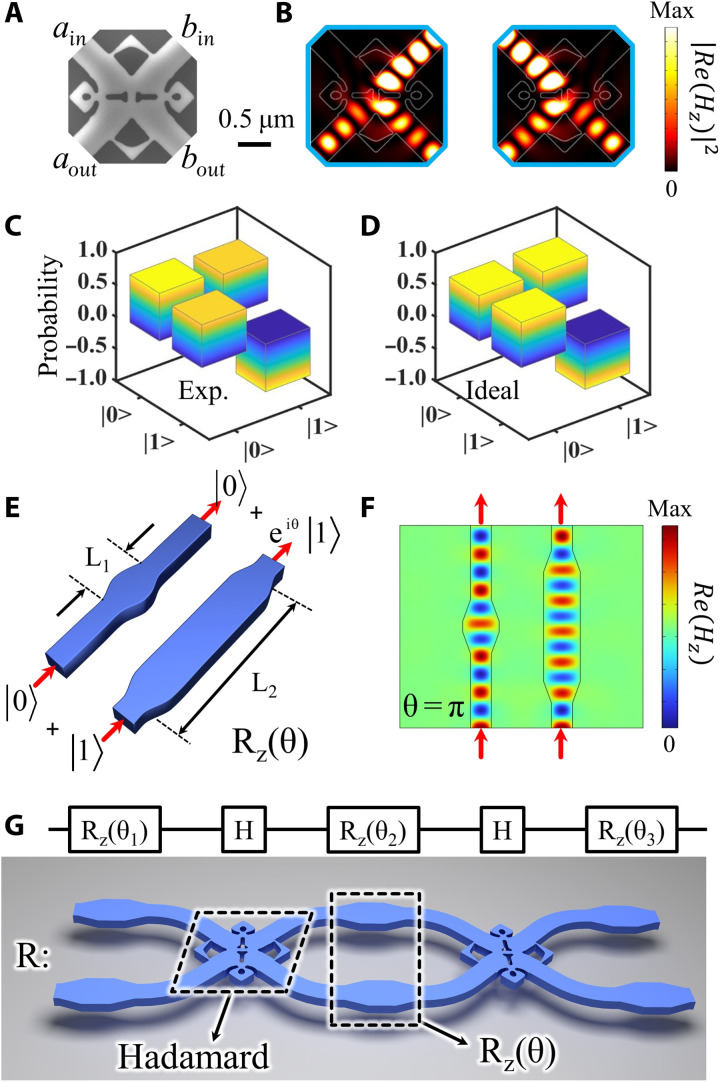
The inverse-designed single-qubit gate. (**A**) Scanning electron microscopy (SEM) image of the Hadamard gate. (**B**) Simulation results of the 50:50 beam splitters of the Hadamard gate. (**C**) Experimental matrix of the Hadamard gate. (**D**) Ideal matrix of the Hadamard gate. (**E**) Phase z gate R_z_(θ) made by the widened waveguide. (**F**) Simulation result of the phase z gate when θ = π. (**G**) Schematic diagram for the arbitrary single-qubit gate consisted of three phase z gates R_z_(θ) and two Hadamard gates.

The optimized gate is fabricated using the electron-beam lithography followed by dry etching, the scanning electron microscopy image of which is shown in Fig. 2A. The detailed fabrication process is described in Methods. It contains four 400-nm-width waveguides (named *a*_in_, *b*_in_, *a*_out_, and *b*_out_, respectively) and an inverse-designed structure. The footprint of the structure is only 1.69 μm^2^ (1.3 μm by 1.3 μm), which is less than one vacuum wavelength. In contrast, the previous works report that the footprints of Hadamard gates made by the directional coupler or MMI are about 10^2^ to 10^3^ μm^2^ (*5*, *7*), which means that the size of the present gate is shrunk two to three orders compared to those of previous works.

The Hadamard operation can be performed in the designed structure based on single-photon interference. When the single-photon state is injected into the waveguide *a*_in_ or *b*_in_, the output photon states become superposition states with a phase difference π/2 at *a*_out_ and *b*_out_. Figure 2B shows the simulated results of field distributions under the single-photon exciting at *a*_in_ or *b*_in_, which indicates that the inverse-designed structure has a nice performance of the 50:50 BS with low loss.

To test whether such a structure can carry out the function of the Hadamard gate, we implement the single-qubit tomography of the gate. In the experiment, the input states of ∣0〉, ∣1〉, $\frac{1}{\sqrt{2}}(|0\rangle +|1\rangle )$, $\frac{1}{\sqrt{2}}(|0\rangle -|1\rangle )$, $\frac{1}{\sqrt{2}}(|0\rangle +i|1\rangle )$, and $\frac{1}{\sqrt{2}}(|0\rangle -i|1\rangle )$ are generated and injected into the Hadamard gate, where ∣0〉 and ∣1〉 represent the quantum states in the waveguide *a*_in_ (*a*_out_) and *b*_in_ (*b*_out_), respectively. After going through the Hadamard gate, the output quantum states are projected to the six states (the same to the input states). Thus, the projection probabilities of these output states for all input states are recorded in a 6 × 6 matrix. On the basis of these measurement data, we retrieve the experimental transformation matrix with the ∣0〉 and ∣1〉 bases, as shown in Fig. 2C. It is clearly seen that the input states for ∣0〉 and ∣1〉 are successfully transformed into $\frac{1}{\sqrt{2}}(|0\rangle +|1\rangle )$ and $\frac{1}{\sqrt{2}}(|0\rangle -|1\rangle )$, respectively. The experimental matrix of the Hadamard operation is very close to the ideal one, which is shown in Fig. 2D. For quantitatively characterizing the Hadamard gate (*46*), we calculate its fidelity *F*_H_ being 0.987(3), which is defined as *F*_H_ = ∣〈ϕ∣*M*_th_*M*_exp_∣ϕ〉∣^2^. Here, *M*_th_ and *M*_exp_ are the theoretical and experimental matrices of the Hadamard gate, and ∣ϕ〉 is defined as quantum state ∣0〉 or ∣1〉. Such a high fidelity further indicates that the function of the Hadamard gate is well implemented.

On the basis of such a Hadamard gate, combined with a phase rotation z gate R_z_(θ), we can construct an arbitrary single-qubit gate R. For the path-encoded scheme, R_z_(θ) is easy to be realized by introducing the phase difference of the photon state between two paths. As shown in Fig. 2E, the phase z gate is constructed by different lengths of the widened waveguides with the width being 700 nm and the lengths being L_1_ and L_2_ (less than 2.5 μm). When L_1_ ≠ L_2_, there is a phase difference θ between the quantum states ∣0〉 and ∣1〉 in these waveguides. Thus, the phase z gate can be realized. To further characterize the quality of R_z_(θ), a special case (the phase θ = π) is simulated and the field distribution is shown in Fig. 2F. The simulation result indicates that the phase z gate has an ultra-low loss and precise phase π attaching to the quantum state ∣1〉. Then, an arbitrary single-qubit gate R is constructed by combining three R_z_ and two Hadamard gates, as shown in Fig. 2G. The phases of three R_z_ are θ_1_, θ_2_, and θ_3_, respectively, which can be adjusted to the fixed values to map the single-qubit state to any point on the Bloch sphere. By the simulation (see section S4 for details), a high performance of the single-qubit gate R is also demonstrated.

### The inverse-designed CNOT gate

**Fig. 3.**
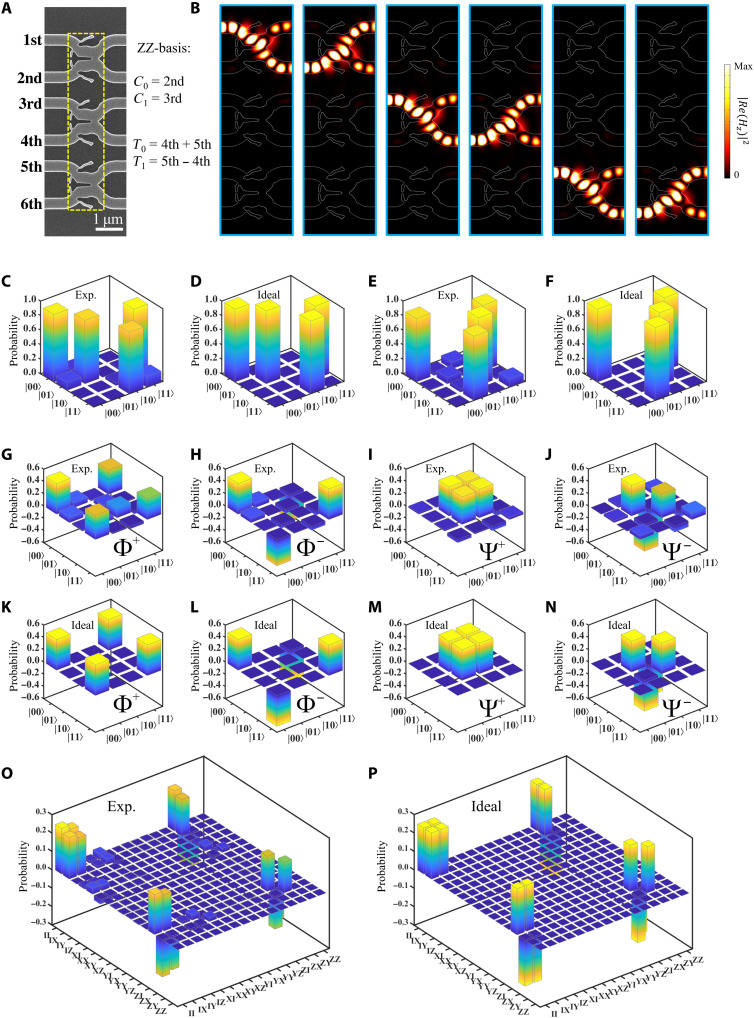
The inverse-designed CNOT gate and its experimental and ideal results. (**A**) SEM image of the CNOT gate. (**B**) Simulation results of three 33:67 beam splitters in the CNOT gate. (**C**) Experimental and (**D**) ideal results of the operation probabilities for the CNOT gate under the ZZ-basis. The experimental fidelity is *F*_ZZ_ = 0.9481 ± 0.0064. (**E**) Experimental and (**F**) ideal results of the operation probabilities for the CNOT gate under the XX-basis. The experimental fidelity is *F*_XX_ = 0.9445 ± 0.0051. (**G** to **J**) Experimental density matrices of four bell states; the fidelities *F*_Bell_ are 0.9034 ± 0.0110, 0.9634 ± 0.0059, 0.9578 ± 0.0068, and 0.9382 ± 0.0067, respectively. (**K** to **N**) Ideal density matrices of four bell states. The quantum process tomography of the CNOT gate for (**O**) the experimental results and (**P**) the ideal results. The process fidelity *F*_CNOT_ is 0.9080 ± 0.0030.

Now, we inverse-design the two-qubit CNOT gate by using the linear optical scheme. Such a scheme has been demonstrated in free space (*2*, *10*) and integrated optics (*4*–*7*, *17*–*20*). The design of the CNOT gate can be realized by combining three 33:67 BSs (the transmittance is *T* = 0.67 and the reflectivity is *R* = 0.33) in parallel. In principle, the target photon is flipped when the two-photon interference happens between the control and target photons. Thus, the inverse design of the CNOT gate first requires the inverse design of the 33:67 BS. The inverse design process of the 33:67 BS is similar to that of the Hadamard gate, except for changing the optimization parameters γ_1_ and γ_2_ in the design process (see section S2 for details). The designed and fabricated CNOT gate is shown in Fig. 3A. The fabrication process of the CNOT gate is also similar to that of the Hadamard gate. The 33:67 BSs are spaced 500 nm apart to ensure that the quantum states inside do not interfere with each other. The width of the CNOT gate is 6.4 μm and the depth is 1.3 μm (less than one vacuum wavelength) in the direction of quantum state propagation. The footprint of the designed CNOT gate (8.32 μm^2^) is the smallest in the world.

In our inverse-designed CNOT gate, there are six input and output waveguides, named from first to sixth, connecting to three 33:67 BSs from top to bottom of Fig. 3A. For simplicity, the quantum states in these input waveguides are defined as ∣1st〉, ∣2nd〉, ∣3rd〉, ∣4th〉, ∣5th〉, and ∣6th〉. Figure 3B shows the simulated field distributions when these single-photon states with λ = 1550 nm are injected into the waveguides, respectively. The devices have a nice performance to implement the function of the 33:67 BSs with low loss and no cross-talk coupling among each other.

To carry out the function of the CNOT gate, we consider the measurement of the operation under **ZZ**-basis and **XX**-basis. The **ZZ**-basis is defined as ∣0_ZZ_〉*_c_* ≡ ∣2nd〉 and ∣1_ZZ_〉*_c_* ≡ ∣3rd〉 for the control qubit, as well as ${|0_{\mathrm{ZZ}}\rangle }_{t}\equiv \frac{1}{\sqrt{2}}(|4\mathrm{th}\rangle +|5\mathrm{th}\rangle )$ and ${|1_{\mathrm{ZZ}}\rangle }_{t}\equiv \frac{1}{\sqrt{2}}(|5\mathrm{th}\rangle -|4\mathrm{th}\rangle )$ for the target qubit. To characterize the operation of this gate, we measure the output for each of the four possible input states: ∣00_ZZ_〉_*c*t_, ∣01_ZZ_〉_*c*t_, ∣10_ZZ_〉_*c*t_, and ∣11_ZZ_〉_*c*t_. The measured results for input-output operation probabilities, normalized by the sum of all coincidence counts obtained for each of the respective input states, are presented in Fig. 3C. The corresponding ideal result is shown in Fig. 3D. By comparison, we find that the experiment result is in good agreement with the theory, indicating a nice performance of the designed CNOT gate. Furthermore, the average transformation fidelity of the CNOT gate can be obtained as *F*_ZZ_ = 0.9481 ± 0.0064, where the definition of this fidelity is $F_{\mathrm{ZZ}}=Tr(\sqrt{\sqrt{M_{\mathrm{th}}}M_{\exp}\sqrt{M_{\mathrm{th}}}})$. Then, let us consider the XX-basis, which is defined as ${|0_{\mathrm{XX}}\rangle }_{c}\equiv$ $\frac{1}{\sqrt{2}}(|2\mathrm{nd}\rangle +|3\mathrm{rd}\rangle )$ and ${|1_{\mathrm{XX}}\rangle }_{c}\equiv \frac{1}{\sqrt{2}}(|2\mathrm{nd}\rangle -|3\mathrm{rd}\rangle )$ for the control qubit, as well as ∣0_XX_〉*_t_* ≡ ∣5th〉 and ∣1_XX_〉*_t_* ≡ ∣4th〉 for the target qubit. As shown in Fig. 3E, the operation of the gate presents the correct output states ∣00_XX_〉_*c*t_, ∣01_XX_〉_*c*t_, ∣10_XX_〉_*c*t_, and ∣11_XX_〉_*c*t_ corresponding to the input states ∣00_XX_〉_*c*t_, ∣10_XX_〉_*c*t_, ∣00_XX_〉_*c*t_, and ∣01_XX_〉_*c*t_, respectively, which is also in good agreement with the theoretical data in Fig. 3F. The average transformation fidelity can be computed as *F*_xx_ = 0.9445 ± 0.0051. Such high fidelities under **ZZ**- and **XX**-basis quantitatively confirm that they implement the quantum CNOT function well. The small discrepancy between the experimental and ideal fidelities is mainly attributed to the inaccuracy of the quantum state preparation and tomography on the chip, and the imperfect BS ratio for the 33:67 BSs. Note that because of the postselection strategy, the success probability of the coincidence measurement for the CNOT gate is theoretically 1/9.

An important function of the CNOT gate is to entangle two quantum states. In a particular case, maximally entangled Bell states Φ^+^, Φ^‐^, Ψ^+^, and Ψ^‐^ can be generated by inputting the superposition states ∣±〉*_c_*∣0〉*_t_* and ∣±〉*_c_*∣1〉*_t_*, where ${|\pm \rangle }_{c}=\frac{1}{\sqrt{2}}({|0\rangle }_{c}\pm {|1\rangle }_{c})$. In the experiment, the corresponding quantum states $\frac{1}{\sqrt{2}}(|2\mathrm{nd}\rangle \pm |3\mathrm{rd}\rangle )$ and $\frac{1}{\sqrt{2}}(|4\mathrm{th}\rangle \pm |5\mathrm{th}\rangle )$ are injected into the CNOT gate. Then, we use the arbitrary single-qubit measurement of the capability to analyze these four output states for performing the quantum state tomography. The phase shifters are adjusted to implement all the measurements in the state preparing and tomography modules of the chip. Therefore, the corresponding density matrix of Bell states can be reconstructed by quantum state process tomography (*47*). Figure 3 (G to J) shows the experimentally measured density matrices of bell states. Correspondingly, the ideal density matrices of bell states are given in Fig. 3 (K to N). It can be seen that all four Bell states are accurately generated. The corresponding fidelities of bell states are obtained from $F_{\mathrm{Bell}}=Tr(\sqrt{\sqrt{\rho_{\mathrm{th}}}\rho_{\exp}\sqrt{\rho_{\mathrm{th}}}})$ as 0.9034 ± 0.0110, 0.9634 ± 0.0059, 0.9578 ± 0.0068, and 0.9382 ± 0.0067, respectively. Here, ρ_exp_ (ρ_th_) is the density matrix reconstructed from the experimental (ideal) data. The results of high fidelities show that four Bell states are well generated by the inverse-designed CNOT gate, which demonstrates the entanglement ability of the gate.

To fully characterize the inverse-designed CNOT gate, we also carry out the quantum process tomography. For a generic quantum process ζ acting on a two-qubit density matrix β, one has $\zeta (\beta )=\sum_{m,n=1}^{15}\chi_{mn}{\hat{A}}_{m}\beta {\hat{A}}_{n}^{†}$, where the operator ${\hat{A}}_{m}$ (${\hat{A}}_{n}$) is defined as the tensor products of Pauli matrices {${\hat{A}}_{m}\equiv \sigma_{i}⊗\sigma_{j}$}, *i*, *j* = 0,…,3, *m* = 0,…,15. Here, the matrix χ*_mn_* contains all the information of the process. The experimentally reconstructed process matrix is plotted in Fig. 3O, which is basically consistent with the ideal case as shown in Fig. 3P. Using the definition of the process fidelity $F_{\exp}=Tr{\left[\sqrt{\sqrt{\chi_{\exp}}\chi_{\mathrm{CNOT}}\sqrt{\chi_{\exp}}}\right]}^{2}/Tr[\chi_{\exp}]Tr[\chi_{\mathrm{CNOT}}]$, we obtain *F*_exp_ = 0.9080 ± 0.0030, which shows a high-performance efficiency of the designed CNOT gate. In addition, our experiment results also show very small imaginary parts (close to zero) for the density matrices of the bell states and the reconstructed process matrix of the CNOT gate. This further demonstrates the nice performance of our CNOT gate. The detailed discussion can be found in section S5.

## DISCUSSION

### The discussion of super-compact quantum circuits

**Fig. 4.**
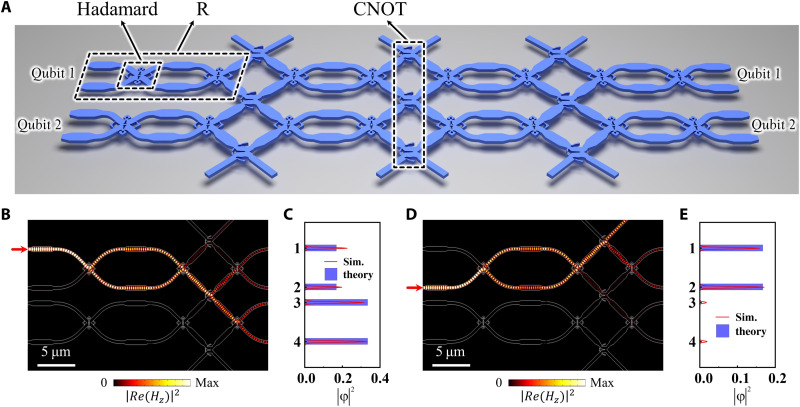
The scheme of inverse-designed super-compact quantum circuits. (**A**) Schematic diagram for inverse-designed super-compact quantum circuits consisted of eight arbitrary single-qubit gates (R) and three CNOT gates. (**B** and **D**) represent the simulation results of the field distribution for the quantum circuit with single-photon exciting from the (B) first and (D) second waveguides. (**C** and **E**) are the comparison figures between theory (blue rectangles) and simulation (red lines) results of the single-photon state probability, where the theory results are calculated by the transfer matrix method and the simulation results are obtained by the field distribution in the waveguides.

One of the major applications of quantum gates is the construction of integrated quantum photonic circuits to implement arbitrary quantum processing. Thus, it is of great significance to demonstrate that our inverse-designed quantum gates can be used to construct such a quantum circuit with a super-compact footprint. As the schematic diagram shown in Fig. 4A, the arbitrary two-qubit quantum circuit consists of three inverse-designed CNOT gates and eight arbitrary R gates. The footprint of such a quantum circuit is approximately 10^3^ μm^2^. It is effectively shrunk four orders of magnitude from ~10^7^ μm^2^ in the previous work (*5*), which indicates that more than 10^4^ two-qubit quantum circuits can be integrated into the same area. The overall size of the proposed quantum circuit can be found in section S6.

In order to test the function of our designed super-compact quantum circuits, we perform a numerical simulation of single-photon state evolution. Figure 4 (B and D) shows squares of probability amplitudes (∣ϕ∣^2^) with the single-photon state exciting from the first and second waveguides, respectively. The corresponding ∣ϕ∣^2^ of output states from the first to the fourth waveguides is marked as the red lines in Fig. 4 (C and E). Meanwhile, we also provide theoretical results for the squares of probability amplitudes of the output superposition state in these four waveguides, which are marked as blue rectangles. The detailed theoretical method is described in section S7. Comparing them, we find that the consistency between the theory and the numerical simulation is very good, which indicates that the circuit has a high performance and a low cross-talk although it is integrated into such a small footprint. Furthermore, the state transform matrices of the quantum circuit are also calculated, and the consistency between the theoretical and numerical results is proved again. This means that quantum chips with good functions can be fabricated by the inverse design method. For some specific two-qubit processes, such as the SWAP gate, a more compact footprint (~50 μm^2^) can be achieved (see section S8 for details).

After two-qubit universal quantum logic gates have been designed, the next milestone goal is to realize the ultra-small quantum computing chip for some certain tasks, such as boson sampling (*48*, *49*). For these tasks, variable optical elements are more meaningful, which is primarily a thermally controlled phase shifter. We believe that it can also be optimized by the inverse design method to obtain ultra-compact footprints. Furthermore, it can greatly reduce the size of tunable quantum chips. In general, the overall fidelity of a quantum circuit decreases with the number of device cascades increasing. Thus, the high fidelity of the unit device is particularly important for large-scale cascaded quantum circuits. Recent works (*50*–*52*) report that single device fidelity of more than 99% enables fault-tolerant quantum computing. In the future, we will take the fidelity as the objective function in the optimization, to obtain high-fidelity quantum gates for large-scale quantum circuits.

Overall, it needs to solve the problems of the full tunability of an optical chip in ultra-small footprints, cross-talks of quantum states in such a small scale, and the fabricated technique of silicon photonic chip with low-loss inverse-designed devices. After these problems are solved, we believe that the ultra-small quantum computing chip can be realized.

In summary, we have designed and fabricated super-compact universal quantum logic gates using the inverse-designed method on the silicon photonic chip with the integrated source. The footprints of CNOT and Hadamard gate are only 8.32 μm^2^ (1.3 μm by 6.4 μm) and 1.69 μm^2^ (1.3 μm by 1.3 μm), respectively. They are the smallest optical quantum gates reported until now. On the basis of these universal quantum logic gates, the silicon photonic quantum circuit to implement arbitrary two-qubit processing has been designed. It is found that the size of the quantum circuit is reduced by four orders of magnitude compared with those previous quantum photonic circuits. The high-performance efficiencies for these super-compact quantum gates and circuits have also been demonstrated. This work provides some previously unknown designs for on-chip integrated quantum information processing, which is expected to solve the scalability problem of optical quantum chips.

## METHODS

### Measurement method

The continuous-wave laser (keysight N7714A) is used to generate the pump light in the experiment, and the wavelengths of the two pump lights are 1538.19 and 1535.04 nm, respectively. The incident laser is first coupled to the single mode fibers (SMFs), combined by the DWDMs, and injected into the chip by the fiber array. Next, the SFWM process is stimulated in two 6-mm-long silicon waveguides, where the frequency-degenerate photon pairs (1536.61 nm) are generated. The two-photon coincidence counts are up to 100 kHz under the pump power of 35 mW. Nine computer-controlled thermal phase shifters are used for generating any biphotonic state and projecting it to any measurement basis. The output photons from the chip couple to the SMFs of the fiber array and go through the DWDMs (filter out the pump light). The output photons are counted by the SNSPDs. Last, we perform a coincidence measurement between the two photons.

The success probability of the quantum logic gate is determined by the collection efficiency of photons, which is constructed by the detection efficiency of photons and the coupling efficiency of the grating coupler. In the experiment, we exactly adjusted the position of the fiber array. The coupling efficiency of the grating coupler can reach −3.5 dB for every coupler at the optimum wavelength. The efficiencies of single photon detectors are about ∼50% and dark count rates are about ∼100 Hz. In the future, we will further increase the collection efficiency to obtain a higher success probability. Potential methods include the utilization of the edge coupling and the SNSPDs with a better performance.

### Sample fabrication

The samples are fabricated using electron beam lithography, followed by dry etching. The substrate is an SOI wafer with a 220-nm-thick top Si layer. ZEP-520A e-beam resist is first spin-coated on the substrate for exposure, and resist patterns are formed after e-beam lithography and development. The fabrication time is about tens of minutes. Then, these resist patterns are transformed to the top Si layer using inductively coupled plasma etching in SF6 and CHF3 gases atmosphere, with ZEP520A used as an etching mask. The etching depth for inverse-designed structures and waveguides is 220 nm. Next, a 1-μm-thick silicon dioxide (SiO_2_) layer is deposited by plasma-enhanced chemical vapor deposition. Last, a layer of 100-nm-thick titanium (Ti) is deposited on top of waveguides to form thermal-optical phase shifters. Moreover, the photolithography tool can also be used to fabricate our device, which can greatly reduce the fabrication time. It is more practical for future large-scale integration. Photon pairs are generated in silicon waveguides with a 450-nm width, 220-nm height, and 6-mm length, by the SFWM nonlinear process. MMIs with a width of 6 μm and a length of 43 μm are used as balanced beamsplitters with a low loss (less than 0.5 dB).

## Supplementary Materials

### This PDF file includes:

Sections S1 to S8 Figs. S1 to S10
