# Supplementary material for: Super-compact universal quantum logic gates with inverse-designed elements
Source: Sci Adv. 2023 May 26;9(21):eadg6685. doi: 10.1126/sciadv.adg6685 (PMC10219593; doi:10.1126/sciadv.adg6685)
Supplement: Supplementary file 1 — Sections S1 to S8 Figs. S1 to S10 [file sciadv.adg6685_sm.pdf]

Science Advances
AAAS

Supplementary Materials for

Super-compact universal quantum logic gates with inverse-designed elements

Lu He *et al.*

Corresponding author: Xiangdong Zhang, zhangxd@bit.edu.cn; Wei Zhang, zwei@tsinghua.edu.cn

Sci. Adv. **9**, eadg6685 (2023)
DOI: 10.1126/sciadv.adg6685

This PDF file includes:

Sections S1 to S8
Figs. S1 to S10

Supplementary Materials for

Super-compact universal quantum logic gates with inverse-designed elements

Lu He[1*], Dongning Liu[2*], Jingxing Gao[2], Weixuan Zhang[1], Huizhen Zhang[1], Xue Feng[2], Yidong Huang[2,3], Kaiyu Cui[2], Fang Liu[2], Wei Zhang[2,3+] and Xiangdong Zhang[1$]

[1]Key Laboratory of advanced optoelectronic quantum architecture and measurements of Ministry of Education, Beijing Key Laboratory of Nanophotonics & Ultrafine Optoelectronic Systems, School of Physics, Beijing Institute of Technology, 100081 Beijing, China.

[2]Frontier Science Center for Quantum Information, Beijing National Research Center for Information Science and Technology (BNRist), Electronic Engineering Department, Tsinghua University, Beijing 100084, China

[3]Beijing Academy of Quantum Information Sciences, Beijing 100193, China.

**These authors contributed equally to this work. [$+]Author to whom any correspondence should be addressed: zhangxd@bit.edu.cn; zwei@tsinghua.edu.cn*

S1. The size of each area in the quantum chip for measuring the CNOT gate.

Here, we take the CNOT gate as an example to show the chip design layout. As shown in Fig. S1a, the whole footprint of the chip is about 4.3mm×1.4mm. It can be seen that most of the chip is the supporting modules to test the super-compact quantum gate.

They correspond to the modules of (I) quantum source, (II) state preparing, (III) quantum gate, and (IV) state tomography, which are mentioned in Fig. 1a of the main text. The size of the quantum source part is about 1400μm×230μm. The size of the state preparing (tomography) part is about 900μm×400μm. The size of the quantum gate part is 1.3μm×6.4μm.

Our proposed super-compact quantum gate is marked in the blue box. The corresponding SEM image and enlarged SEM image can be seen in Figs. S1b and S1c. We have demonstrated that our quantum gate has a good performance through traditional devices (MZI, quantum source, etc.).

Through Fig. S1a, we can clearly compare the size of the inverse-designed quantum gate with that of traditional quantum devices. The size of traditional quantum devices is much larger than that of inverse-designed quantum devices. We expect to apply inverse-designed devices on the entire chip in the future, for reducing the overall size of quantum chips.

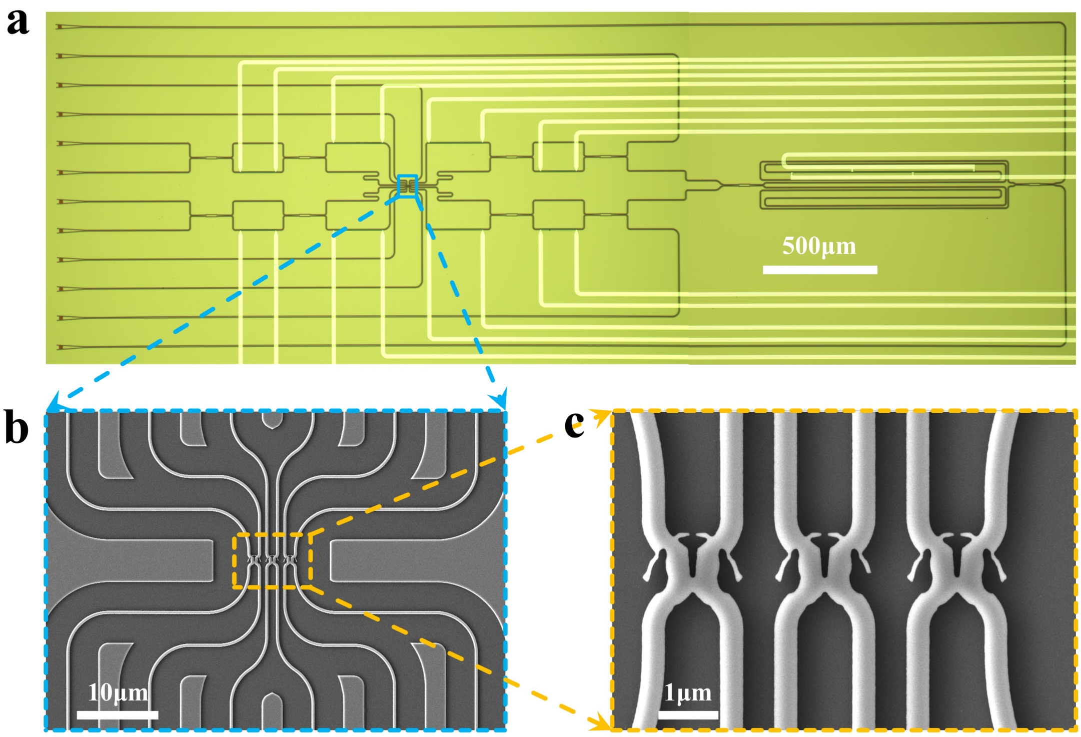

Fig. S1. The quantum chip for measuring the CNOT gate. a. The microscope picture of the quantum chip, which corresponds to Fig. 1a in the main text. Because the microscope field of view is relatively small, the whole microscope picture is taken by twice independent photographing. And then we joint them together. **b.** The SEM image of the CNOT gate and its connected waveguide. **c.** The enlarged SEM image of the CNOT gate.

S2. The detailed optimization procedures of Hadamard and CNOT gates.

From the main text, it is known that the kernels of the Hadamard and CNOT gates are mainly formed by the 50:50 and 33:67 beam splitters for the linear optical scheme. Thus, in this section, we focus on the optimization of the 50:50 and 33:67 beam splitter in a single structure, where a pair of input ports and two output ports (named a_{in}, b_{in} and a_{out}, b_{out} ports) exist, as shown in Fig. S2a. Note that in our optimization process, the squares of the amplitude of the single photon quantum state corresponds to the optical field energy.

For the 50:50 beam splitter, if only one single photon is injected into the system from any input port (a_{in} or b_{in}), the output photonic quantum state could become the superposition at two output ports (a_{out} and b_{out}) with equal squares of amplitudes at the same time. To fulfill these operations, the system must possess a mirror symmetry with respect the dashed line (called mirror symmetry line), as marked in Fig. S2a. In this case, only a half region is needed to be optimized where the other mirror symmetric part should have the same dielectric distribution.

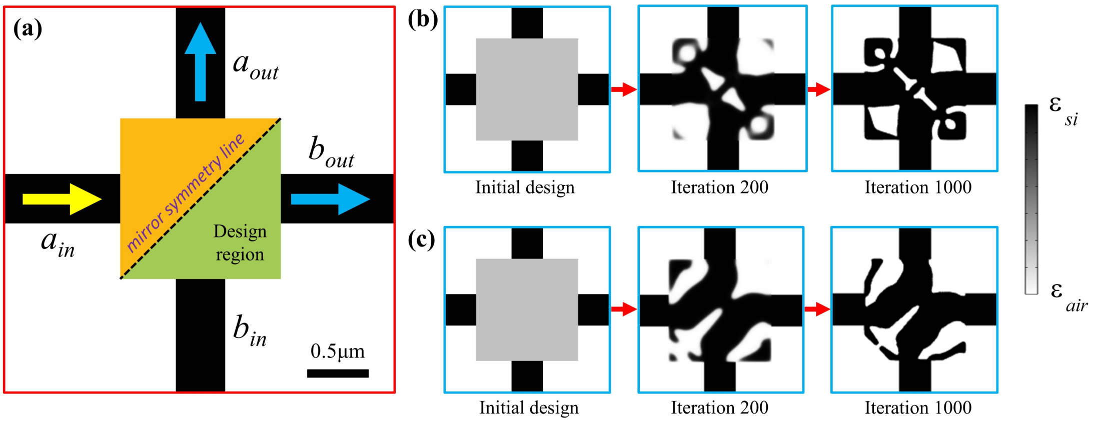

Fig. S2. The inverse design process of the 50:50 and 33:67 beam splitters. (a) the schematic diagram of the optimization model. The designable region (green triangle) and the mirror symmetry line (dashed line). The inverse design processes of (b) 50:50 and (c) 33:67 beam splitters. The initial design is an intermediate material between Si and SiO_2. With the optimization going on, the materials in the design region tend to binarization. The final design is determined by 1000 iterations.

To achieve the above-mentioned device, the associated objective function expressed as Eq. (1) in the main text is carried out. The detailed material parameters (the permittivities of SiO_2 and Si layer) are set as ε_{sio_2}=2.1 and ε_{si}=12.1. For the additional condition $\gamma_1 \le c_1^2/c_2^2 \le \gamma_2$, we set γ_1 =0.9 and γ_2=1.1 in the optimization process. In this way, the inverse design process is carried out from the initial design of the homogeneous material distribution with the dielectric constant being $\varepsilon=(\varepsilon_{sio_2}+\varepsilon_{si})/2$. The intermediate structures generated by the inverse design process are shown in Fig. S2b. With the increasing of iteration steps, the objective function is gradually enlarged and the final structure with the optimal performance appears after 1000 iterations.

For the 33:67 beam splitter, the inverse design process is similar to that of the 50:50 beam splitter, except for adjusting the parameters γ_1 and γ_2 in the extra condition. They are changed as γ_1 =0.62 and γ_2=0.72. Similarly, the intermediate structures generated by the inverse design process are shown in Fig. S2c. The corresponding final structure appears after 1000 iterations. Except for these limiting conditions, the linear material interpolation, projection, and filtering procedures are used in the inverse design process.

Finally, the structures of the optimized 50:50 and 33:67 beam splitters are exported for realizing the Hadamard and CNOT gates, respectively.

S3. The detailed discussion of the sensitivity analysis of the device performance.

In optical chip fabrication, the most common defect is the manufacturing deviation. Generally, there is an error of 20nm in the e-beam lithography etching, due to the instability of the dose of the electron beam. Thus, the designed device can be used for experimental fabrication if it can tolerate a manufacturing defect of under (over) etching with ±10nm.

Here, we consider the sensitivity analysis of the device performance with some manufacturing defects. Such an analysis includes two aspects: the first one is that we used the double filtering method (Refs. [45, 46] in the main text) in the inverse-design process, which can increase the robustness of the device; the second one is that we also did a simulation to verify the performance of our device. Let us introduce them in detail:

1) In order to increase the robust property against manufacturing defects, we use the double filtering method in the inverse-design process. In brief, the double filtering method consists of applying the filter and threshold procedure twice on the design field, where in the second application three different threshold values are applied to obtain three different realizations of the design fields corresponding to under (over) etching. So, the over and under etching cases are simultaneously optimized. In such a way, we can get a robust device against the manufacturing defect of under (over) etching, which is the most common defect in optical chip fabrication. Thus, the inverse-designed structure possesses the robust property against manufacturing defects in a certain extent.

2) After getting the inverse-designed structure from the optimization process, we further verify the device performance against manufacturing defects by the simulation. Here, we get three structures from the optimization results, which are exact etching (0nm) case and over or under etching (±10nm) cases. As shown in Fig. S3a, we selected 33:67 beam splitter as an example to perform sensitivity analysis. In the enlarged view of Fig. S3b, we can clearly see that the over or under etching (±10nm) is considered in the design layout. And then, we do the simulation (Figs. S3c-S3e) with the error of ±10nm. In these errors, the main function of the device (beam splitting ratio) deteriorate from 34:66 to 30:70 at the wavelength of 1550nm. The ideal beam splitting ratio is 33:67. The fidelity of the CNOT gate deteriorate from 99.96% to 99.36% in the theoretical calculation. A similar analysis about the required range of the splitting ratio can be found in Ref. [7] of the main text.

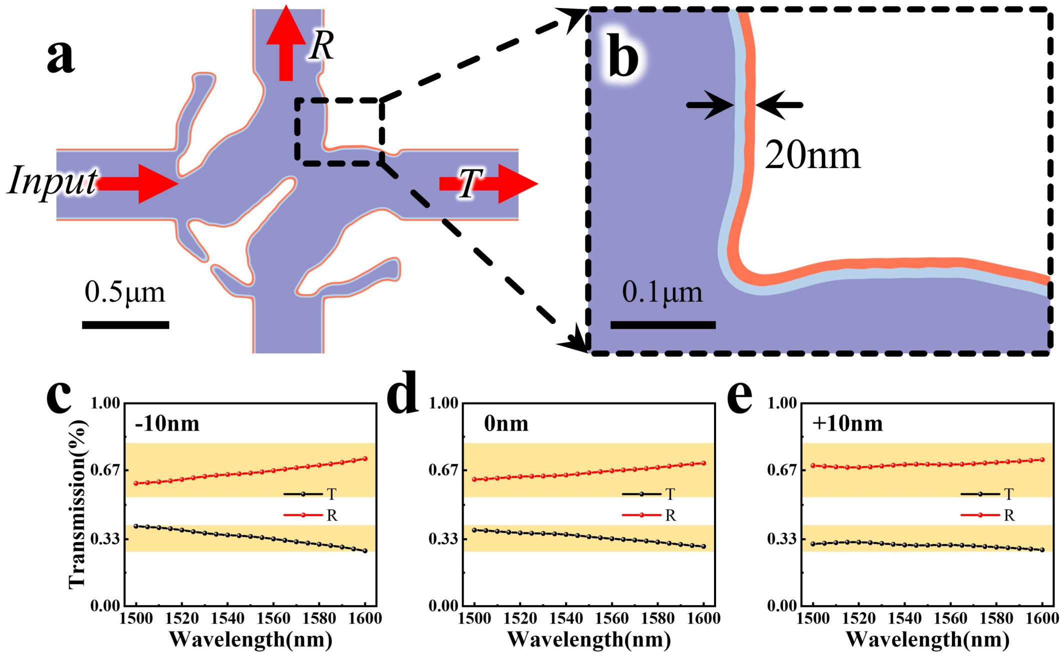

Fig. S3. The sensitivity analysis of our designed device. (a) The design layout with over (under) etching (±10nm). (b) The enlarged view of (a). The simulated results of the 33:67 beam splitter with (c) over etching (-10nm), exact etching, and under etching (+10nm). The yellow region represents the usable range of splitting ratio for the CNOT gate. For T, the range is from 0.60 to 0.73. For R, the range is from 0.27 to 0.40.

S4. The detailed discussion of the arbitrary single qubit gate.

The single qubit gate consists of the Hadamard gate and the phase z gate. To ensure that their quantum circuits cascaded by individual gates work well, we must consider that the operating frequency range of these designed devices can cover each other.

First, we study the operating frequency range of the phase z gate by the simulation. We fix L_2=0.1μm and calculate the phase difference between the two waveguides after the gate by sweeping the parameter L_1, as shown in Fig. S4. It can be seen that in the whole communication band (1500-1600nm), the phase z gate can generate a stable phase difference θ by adjusting L_1.

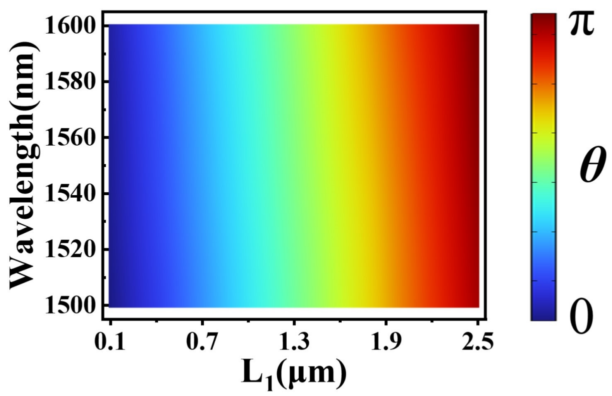

Fig. S4. The additional phase difference of the phase z gate by adjusting L_1.

And then, we study the theoretical model and the numerical simulation of the arbitrary single qubit gate R, as mentioned in Fig. 2g of the main text. For the path-encoded scheme, the R gate can be represented the product as a series of matrices. Thus, the quantum state evolution can be expressed as follow:

$$\left|\varphi_{out}\right\rangle = \frac{1}{2}\begin{pmatrix}1 & \\ & e^{i\theta_3}\end{pmatrix}\begin{pmatrix}1 & 1\\ 1 & -1\end{pmatrix}\begin{pmatrix}1 & \\ & e^{i\theta_2}\end{pmatrix}\begin{pmatrix}1 & 1\\ 1 & -1\end{pmatrix}\begin{pmatrix}1 & \\ & e^{i\theta_1}\end{pmatrix}\left|\varphi_{in}\right\rangle. \tag{S1}$$

When a quantum state $\left|\varphi_{in}\right\rangle$ is injected into the single qubit gate R, any unitary transformation can be acted on it by adjusting the phase of θ_1, θ_2, and θ_3. Now, we give three examples to demonstrate its function through the numerical simulations. In these three cases, the fixed phases in R gates are determined as $\theta_1=\pi$, $\theta_2=\pi/2$, and $\theta_3=0$, respectively. Hence, based on Eq. (S1), the output qubits a, b, and c become $\left|0\right\rangle$, $\frac{1}{\sqrt{2}}(\left|0\right\rangle + i\left|1\right\rangle)$, and $\left|1\right\rangle$, where $\left|0\right\rangle$ and $\left|1\right\rangle$ are the quantum state in the different paths. As shown in Figs. S5a-S5c, these three cases are simulated with the single photon exciting from the upper waveguide, where the quantum state is defined as $\left|0\right\rangle$. Correspondingly, the quantum state in the lower waveguide is $\left|1\right\rangle$. We find that the output quantum states indeed become the expected states after going through the R gate. In Fig. S5d, we mark these output quantum states to three points on the Bloch sphere. By the way, an arbitrary output quantum state can also be generated by the R gate, corresponding to the arbitrary point on the Bloch sphere. So, the simulation results possess a nice consistency with the theory results. It indicates that the R gate has a high performance even though that is integrated into such a small footprint. The overall size

of the single-qubit quantum circuit in Fig. S5a-S5c is about 20μm×10μm.

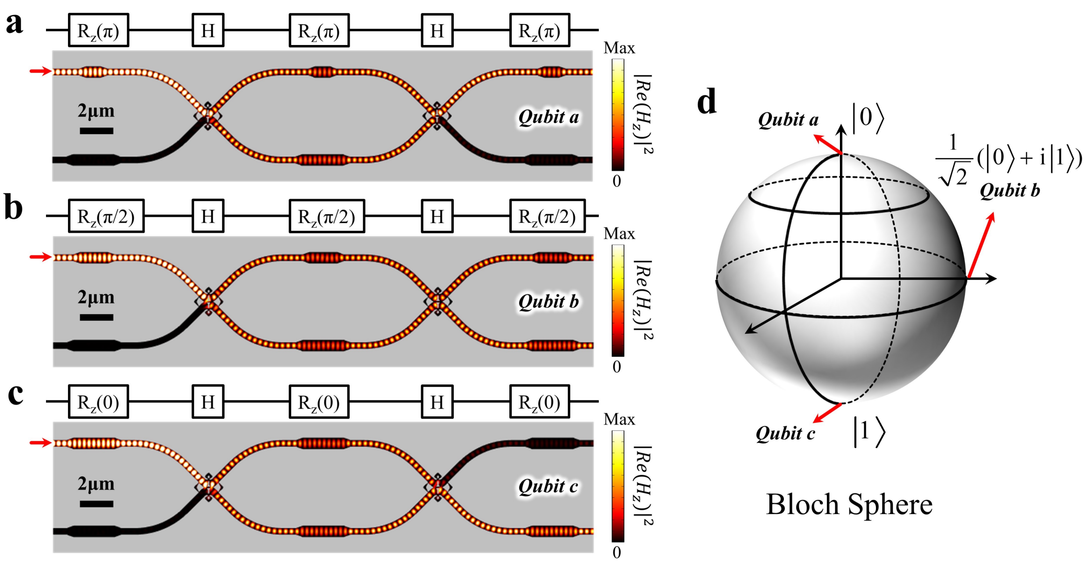

Fig. S5. The simulation results of the arbitrary single qubit gate. The phases of the phase z gates are set as (a) π, (b) π/2, (c) 0. (d) The Bloch Sphere of the single qubit gate R. The corresponding output qubits are marked on the Bloch Sphere.

Finally, let us explain the reason for the ultra-compact footprint of the cascaded R gate. In previous works, the quantum circuits are constructed by optical devices with large footprints and some connected waveguides on chips. The length of the waveguides is on the same order of magnitude with the size of optical devices. Due to the fabrication error, the phase accumulative effect causes an unstable phase difference between these waveguides. Thus, an additional heat electrode is indispensable to correct the phase difference between the path-encoded waveguides. The large optical devices and the connected waveguides with electrodes cover an enormous footprint with the scale of millimeters in traditional schemes. It brings about a great disadvantage for large-scale integrations. However, for our inverse-designed gate with a supercompact footprint (less than a wavelength), the length of the corresponding connected waveguide is about several micrometers. In this scale, the accumulative phase is so small that can be ignored. Thus, the R gate can be integrated into such a small area. Furthermore, the quantum circuits can be constructed by the R gates and the CNOT gates. In general, the size of quantum circuits can be effectively reduced from ~10^7μm^2 in the previous work to ~10^3μm^2 by using our inverse-designed quantum gates. The footprint is shrunk 4 orders of magnitude. The supercompact footprint of our devices indicates that

more than 10^4 quantum circuits can be integrated into the same area. For modern fabrication technology of the photonic chip, such a 2-qubit circuit (Ref. [5] of the main text) can be implemented based on the basic logic components of Hadamard and CNOT gates.

S5. The imaginary parts for the density matrices of the bell states and the reconstructed process matrix of the CNOT gate.

To completely characterize the CNOT gate and its generated bell states, we plot the imaginary parts for the density matrices of the bell states and the reconstructed process matrix of the CNOT gate, as shown in Fig. S6. It is seen that these imaginary parts can be limited to very small values (close to zero). Thus, we believe our inverse-designed CNOT gate possess a high performance.

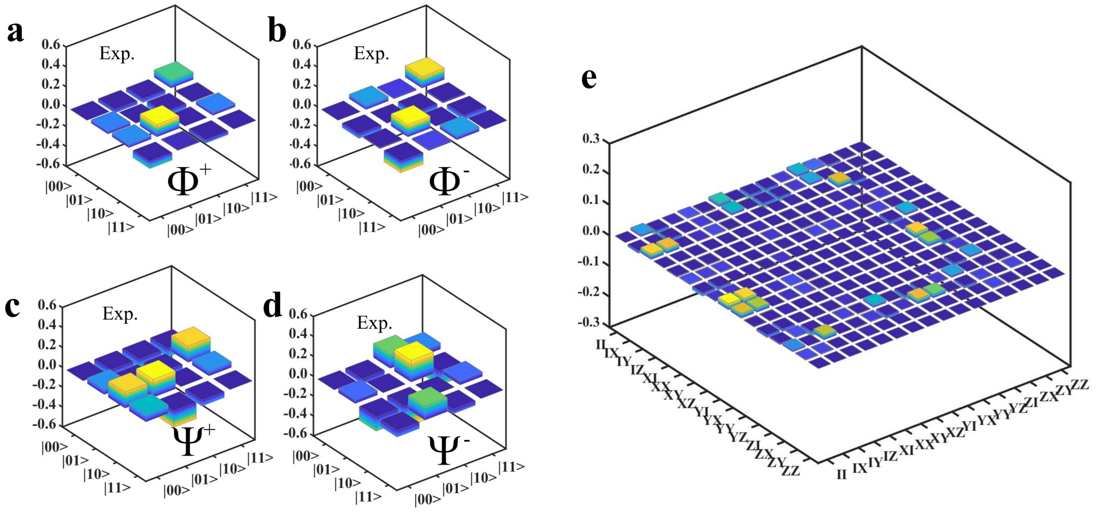

Fig. S6. The experimental results of the imaginary parts for the reconstructed matrices. The imaginary parts of the density matrices of the bell states (a) Φ^+, (b) Φ^-, (c) Ψ^+, and (d) Ψ^-. (e) The imaginary part of the reconstructed process matrix of the CNOT gate.

S6. The overall size of the proposed quantum circuit.

Here, we also show the overall size of the design layout for our proposed inverse-designed quantum circuit in Fig. S7a. This corresponds to the theoretical model shown in Fig. S7b. The overall size of the quantum circuit in Fig. 4 of the main text is about 100μm×25μm.

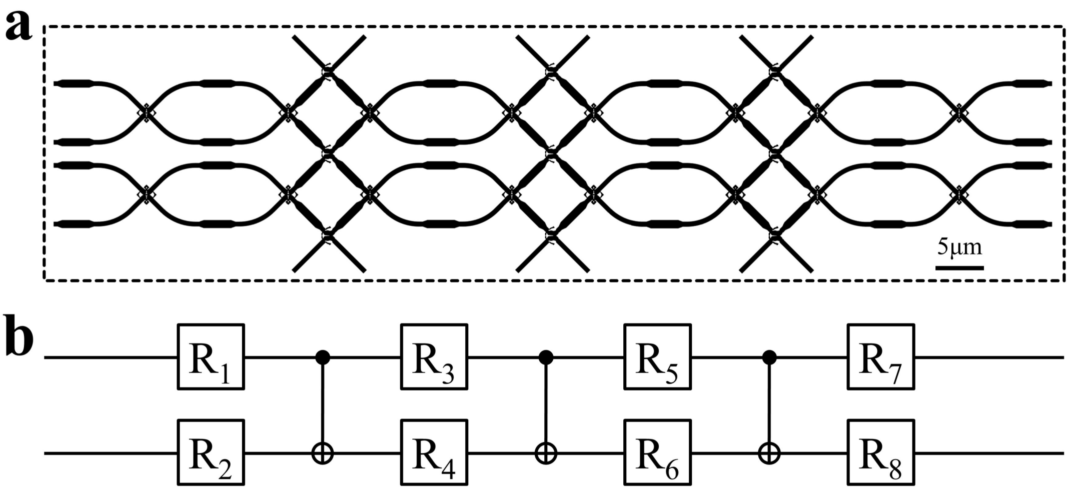

Fig. S7. The 2-qubit quantum circuit by inverse-designed quantum gates. (a) the design layout of the 2-qubit quantum circuit. (b) The corresponding theoretical model of the quantum circuit.

S7. The simulation details of the optical transform matrix.

In Figs. 4b and 4d of the main text, we study the evolution process of the quantum state in the quantum circuit with the single photon exciting. The comparison figures of simulation and theory results are plotted in Figs. 4c and 4e of the main text. Next, we can also obtain the total optical matrix of the quantum circuit. Here, we mainly introduce the transform matrix method to obtain the theory results.

First, we build the transform matrices, M_1, M_2, and M_3, of these optical devices. Their expressions are

$$M_1 = \begin{pmatrix} r_1 & it_1 & & & & \\ it_1 & r_1 & & & & \\ & & r_1 & it_1 & & \\ & & it_1 & r_1 & & \\ & & & & r_1 & it_1 \\ & & & & it_1 & r_1 \end{pmatrix}, \tag{S2}$$

$$M_2 = \begin{pmatrix} 0 & & & & & \\ & r_2 & it_2 & & & \\ & it_2 & r_2 & & & \\ & & & r_2 & it_2 & \\ & & & it_2 & r_2 & \\ & & & & & 0 \end{pmatrix}, \tag{S3}$$

$$M_3 = \begin{pmatrix} 0 & & & & & \\ & 1 & & & & \\ & & e^{i\theta_1} & & & \\ & & & 1 & & \\ & & & & e^{i\theta_2} & \\ & & & & & 0 \end{pmatrix}, \tag{S4}$$

where M_1 is the transform matrix of the CNOT gate with r_1 (t_1) being $1/\sqrt{3}$ ($\sqrt{2/3}$). M_2 is the transform matrix of the Hadamard gate with r_1 (t_1) being $1/\sqrt{2}$. M_3 is the transform matrix of the phase z gate with θ_1(θ_2) being a fixed phase. Next, we have the quantum state evolution equation:

$$|\varphi_{out}\rangle = M_3 M_2 M_3 M_2 M_3 M_1 M_3 M_2 |\varphi_{in}\rangle \tag{S5}$$

where $|\varphi_{out}\rangle$ ($|\varphi_{in}\rangle$) is the output (input) quantum state.

For simplicity, we set the phases of the phase z gates θ_1 and θ_2 as 0. The input states $|\varphi_{in}\rangle$ are set as $(0,1,0,0,0,0)^T$ and $(0,0,1,0,0,0)^T$, corresponding to Figs. 4b and 4d in the main text, respectively. By the calculation of Eq. (S5), the square of probability amplitude $|\varphi|^2$ of the output quantum state become $(0,1/6,1/6,1/3,1/3,0)^T$ and $(0,1/6,1/6,0,0,0)^T$, which are plotted in Figs. 4c and 4e (blue rectangle) in the main text. The theory results possess a nice consistency with the simulation results (marked as the red lines).

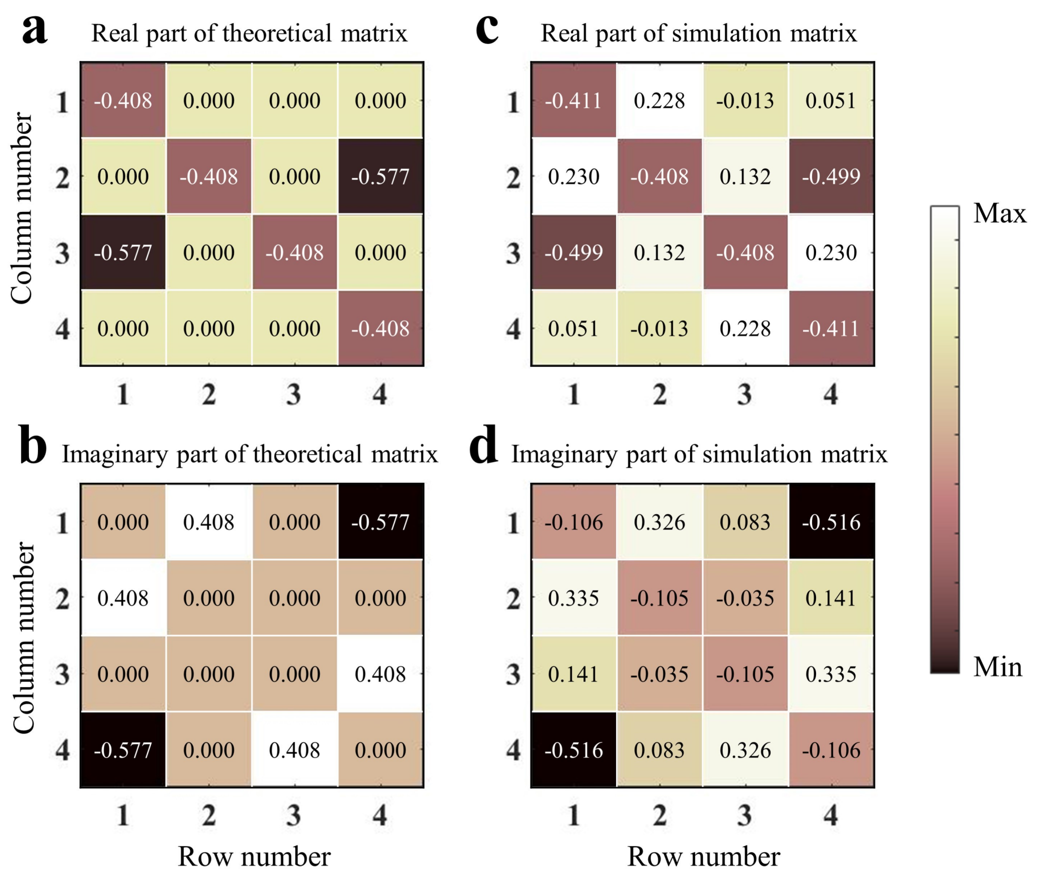

Fig. S8. The total optical transform matrix of the quantum circuit. a. The real part of the theoretical matrix. b. The imaginary part of the theoretical matrix. c. The real part of the simulation matrix. d. The imaginary part of the simulation matrix.

Moreover, we can also calculate the state transform matrices of the quantum circuit by theory and simulation methods. The theoretical transform matrix is obtained from Eq. (S5). The real and imaginary parts of the matrix are plotted in Figs. S8a and S8b. The simulation matrix elements are extracted from the quantum states from the output waveguides (1st-4th) in the full-wave simulation of the quantum circuit. The corresponding real and imaginary parts of the simulation matrix are plotted in Figs. S8c and S8d. Finally, we present the simulation results from our design, compared with the theoretical results, revealing an excellent agreement. It indicates that the circuit has high performance and can perform its function well.

S8. The inverse-designed SWAP gate.

Here, we provide an implementation scheme for the specific 2-qubit gate, the SWAP gate. It consists of four Hadamard gates and three CNOT gates, as shown in Fig. S9a. In this way, a more compact footprint (~50μm^2) of the SWAP gate can be achieved. The encoding scheme of the SWAP gate is same to the CNOT gate in the main text.

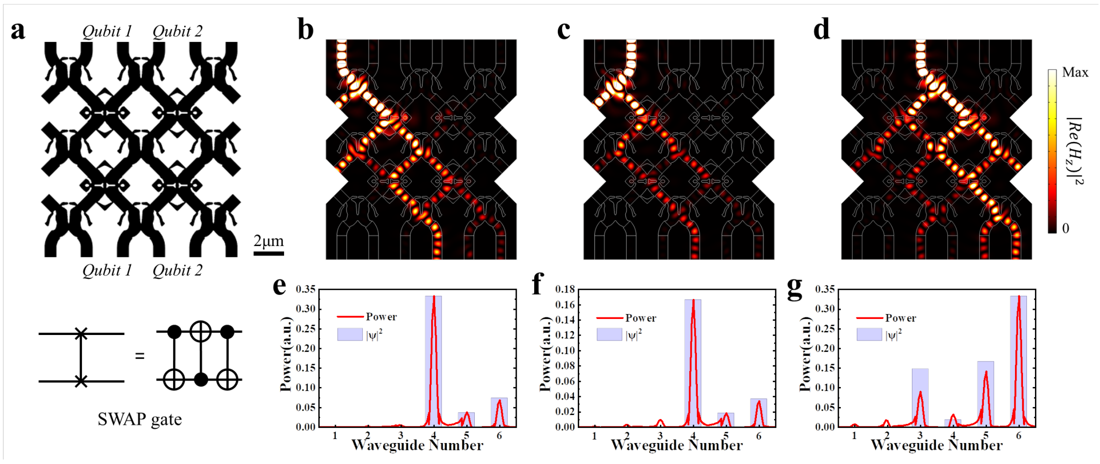

Fig. S9. The inverse-designed SWAP gate. a. The layout structure of the inverse-designed SWAP gate. **b-d.** The simulation results of the SWAP gate with the single photon state exciting. **e-g.** The comparison of the theory (blue rectangle) and simulation (red line) cases, corresponding to **b-d**, respectively.

And then, in order to demonstrate the function of the SWAP gate, we simulate the situation with a single photon exciting. The quantum state is injected in the 1st, 2nd, and 3rd waveguides, and the evolution processes are shown in Figs. S9b-S9d. Due to the symmetry of the SWAP gate, the evolution processes of the input quantum states exciting at the 4th, 5th, and 6th waveguides are same to the above cases. In order to test the function of the SWAP gate, we perform a numerical simulation of single-photon state evolution. Figs. S9b-S9d show squares of probability amplitudes ($|\varphi|^2$) with the single-photon state exciting from the 1st to 3rd waveguides, respectively. The corresponding $|\varphi|^2$ of output states from the 1st to 6th waveguides are marked as the red lines and shown in Figs. S9e-S9g.

On the other hand, for the theoretical description, we can also build the transform matrices. The quantum state evolution equation is

$$|\varphi_{out}\rangle = M_1M_2M_1M_2M_1|\varphi_{in}\rangle \tag{S6}$$

where $|\varphi_{out}\rangle$ ($|\varphi_{in}\rangle$) is the output (input) quantum state of the SWAP gate. The input states $|\varphi_{in}\rangle$ are set as $(1,0,0,0,0,0)^T$, $(0,1,0,0,0,0)^T$, and $(0,0,1,0,0,0)^T$, corresponding to Figs. S9b, S9c, and S9d, respectively. By the calculation of Eq. (S6), the square of probability amplitude $|\varphi|^2$ of the output quantum state become $(0,0,0,0.333,0.037,0.074)^T$, $(0,0,0,0.167,0.019,0.037)^T$ and $(0,0,0.148,0.019,0.167,0.333)^T$, which are plotted in Figs. S9e-S9g (blue rectangle). The square of probability amplitude $|\varphi|^2$ of the quantum state is plotted with the theory (blue rectangle) and simulation (red line) case, as shown in Figs. S9e-S9g.

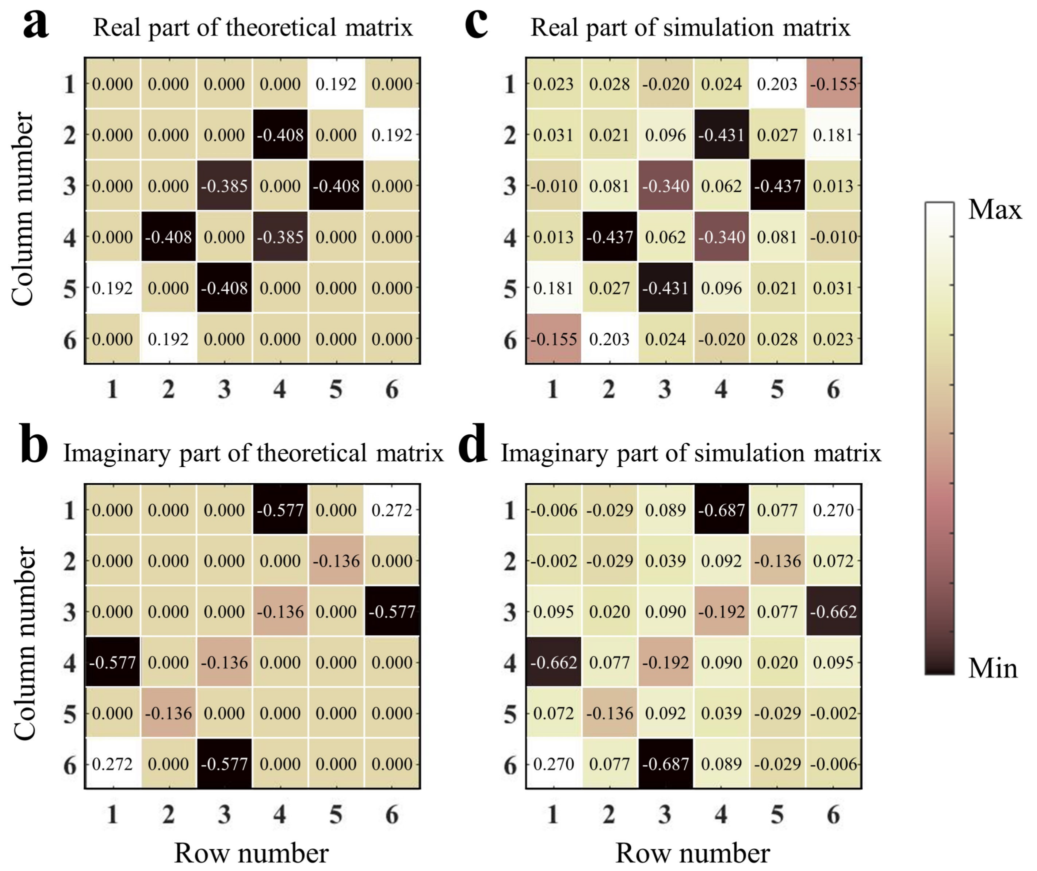

Fig. S10. The total optical transform matrix of the SWAP gate. a. The real part of the theoretical matrix. b. The imaginary part of the theoretical matrix. c. The real part of the simulation matrix. d. The imaginary part of the simulation matrix.

Moreover, we can also calculate the transform matrices of the SWAP gate by the theory and

the simulation methods. The theoretical transform matrix is obtained from Eq. (S6). The real and imaginary parts of the matrix are plotted in Figs. S10a and S10b. The simulation matrix elements are extracted from the quantum states from the output waveguides (1st-6th) in the full-wave simulation of the SWAP gate. The corresponding real and imaginary parts of the simulation matrix are plotted in Figs. S10c and S10d. By comparison, we find that the theory results possess a nice consistency with the simulation results. It indicates that the circuit has a high performance and a low loss even though that is integrated into such a small footprint (~50μm^2). Thus, we believe our inverse-designed SWAP gate can perform its function well.
